# A Large-Scale COVID-19 Twitter Chatter Dataset for Open Scientific Research—An International Collaboration

**DOI:** 10.3390/epidemiologia2030024

**Published:** 2021-08-05

**Authors:** Juan M. Banda, Ramya Tekumalla, Guanyu Wang, Jingyuan Yu, Tuo Liu, Yuning Ding, Ekaterina Artemova, Elena Tutubalina, Gerardo Chowell

**Affiliations:** 1 Department of Computer Science, Georgia State University, Atlanta, GA 30303, USA; rtekumalla1@student.gsu.edu; 2 Missouri School of Journalism, University of Missouri, Columbia, MO 65201, USA; gwnd6@mail.missouri.edu; 3 Department of Social Psychology, Universitat Autònoma de Barcelona, 08035 Barcelona, Spain; jingyuan.yu@e-campus.uab.cat; 4 Department of Psychology, Carl von Ossietzky Universität Oldenburg, 26129 Oldenburg, Germany; tuo.liu@uol.de; 5 Language Technology Lab, Universität Duisburg-Essen, 47057 Duisburg, Germany; yuning.ding@uni-due.de; 6 Faculty of Computer Science, Higher School of Economics—National Research University, 101000 Moscow, Russia; ktr.che@me.com; 7 Faculty of Chemistry, Kazan Federal University, 420008 Kazan, Russia; ElVTutubalina@kpfu.ru; 8 Department of Population Health Sciences, Georgia State University, Atlanta, GA 30303, USA; gchowell@gsu.edu

**Keywords:** public datasets, open science, COVID-19, social media, data sources

## Abstract

As the COVID-19 pandemic continues to spread worldwide, an unprecedented amount of open data is being generated for medical, genetics, and epidemiological research. The unparalleled rate at which many research groups around the world are releasing data and publications on the ongoing pandemic is allowing other scientists to learn from local experiences and data generated on the front lines of the COVID-19 pandemic. However, there is a need to integrate additional data sources that map and measure the role of social dynamics of such a unique worldwide event in biomedical, biological, and epidemiological analyses. For this purpose, we present a large-scale curated dataset of over 1.12 billion tweets, growing daily, related to COVID-19 chatter generated from 1 January 2020 to 27 June 2021 at the time of writing. This data source provides a freely available additional data source for researchers worldwide to conduct a wide and diverse number of research projects, such as epidemiological analyses, emotional and mental responses to social distancing measures, the identification of sources of misinformation, stratified measurement of sentiment towards the pandemic in near real time, among many others.

## 1. Introduction

The first cases of COVID-19 pandemic were first identified of a cluster of viral pneumonia patients of unknown etiology in the city of Wuhan, China, in December 2019. Unfortunately, interventions to contain its spread were not implemented rapidly enough to limit the spread of the virus to China’s borders. While transmission has been dramatically reduced in China through strict social distancing interventions, the virus was exported to multiple countries and is now generating sustained transmission in multiple areas of the world, including areas with active hotspots of the disease including the United States, Italy, Spain, and France [1]. As of 1 July 2021, 183,200,380 global cases have been recorded including 3,966,198 deaths according to the worldometer coronavirus pandemic tracker [2].

While the ongoing COVID-19 pandemic has presented unprecedented challenges to humanity, the wider scientific community can only advance science with access to openly available data. Social media platforms such as Twitter and Facebook contain an abundance of text data that can be utilized for research purposes. Over the last decade, Twitter has proven to be a valuable resource during disasters for many-to-many crisis communication [3,4,5]. With Twitter data, researchers have shown that it is possible to analyze symptom configurations, risk factors, origin, virus genetics, and spread patterns that can be studied and monitored [6,7,8,9]. Recent studies [10,11] prove that data sharing improves quality and strengthens research, with collaborative efforts providing an opportunity for researchers to continually enhance research ideas and avoid redundant efforts [12,13].

We started to release our data to the public for the greater good when the dataset accumulated 40 million tweets on 23 March 2020 [14]. Since then, we have been providing updates every two days [15] and a cumulative update every week, most recently on 27 June 2021 [16]. The latest full dataset update had over 1,122,879,197 tweets available for researchers. The community response by word of mouth has led to over 97,921 views and over 127,956 downloads of the resource. Moreover, several international researchers have reached out to contribute data and provide analysis expertise. Such engagement shows the value of this kind of data and that scientists want to come together to create extensive resources for the benefit of society. Aside from providing the full dataset with retweets included, we provide a clean version with no retweets for researchers with limited resources to access a lighter version of the dataset. Furthermore, to assist researchers for NLP tasks, we provide the top 1000 frequent terms, 1000 bigrams, and 1000 trigrams. The released dataset adheres with FAIR principles [17]. Due to Twitter’s terms of service, tweet text cannot be shared. Therefore, tweet IDs are publicly made available using Zenodo [18]. Tweet IDs can be hydrated using tools such as Social Media Mining Toolkit or twarc [19,20]. The dataset deliverables [15,18] include tweet identifiers, tweet language, and code to process the tweets. Please note that the code to process the tweets requires the tweets to be hydrated. We also provide the date and time meta-data elements of our dataset to allow groups to narrow their research questions to certain days and avoid having to hydrate the whole resource at once. Finally, we are also welcoming any additional data that provide new tweets to this resource.

## 2. Materials and Methods

The initial versions of this dataset [14,21] only included data collected from the publicly available Twitter Stream API with a collection process that gathered any available tweets within the daily restrictions from Twitter from January to 11 March, filtering them on the following 3 keywords: “coronavirus”, “2019nCoV”, ”corona virus”. We shifted our focus to exclusively collect COVID-19 tweets on 12 March 2020 with the following keywords: “COVD19”, “CoronavirusPandemic”, “COVID-19”, “2019nCoV”, “CoronaOutbreak”, “coronavirus”, “WuhanVirus”, thus the number of tweets gathered dramatically expanded the dataset. Please note that the Stream API only allows free access to a one percent sample of the daily stream of Twitter. Our methodology relies on Python and the Tweepy package [22], as in our previous work [23]. We recently received another set of 30+ million tweets collected from 27 January 2020 to 27 March 2020 from our co-author, Jingyuan Yu, and his collaborators with the following keywords: “coronavirus”, “wuhan”, “pneumonia”, “pneumonie”, “neumonia”, “lungenentzündung”, “COVID19”. These tweets were collected in the following languages: English, French, Spanish, and German, while our original collection was performed for any language available. We fully integrated and deduplicated our collaborators’ tweet collection with ours, thus the numbers and tweets presented in this dataset are of unique tweet identifiers from 1 January 2020 to 27 June 2021 (at the time of writing). In version 10, we added ~1.5 million tweets in the Russian language collected between 1 January and 8 May, graciously provided to us by our co-authors Ekaterina Artemova and Elena Tutubalina. 

**Figure 1:**
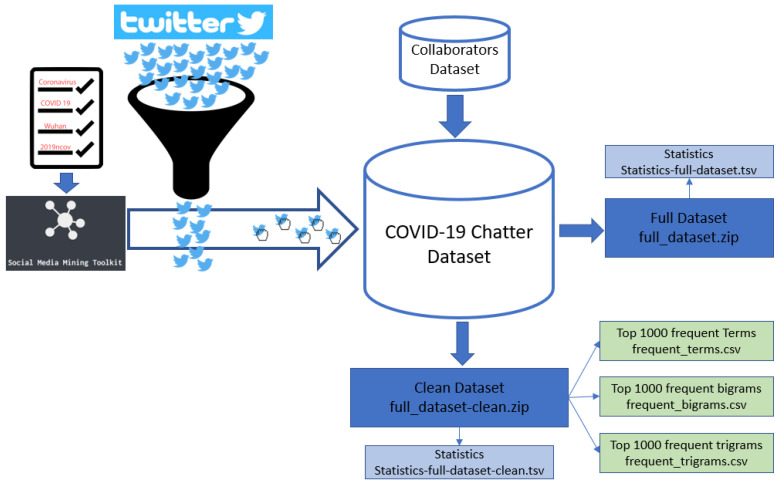
Dataset gathering and construction steps.

As previously mentioned, the number of collected tweets tremendously increased since starting a dedicated collection. All our preprocessing scripts utilize components of the Social Media Mining Toolkit (SMMT) [19]. We make a distinction between our full and clean versions of the dataset. The full dataset consists of both tweets and retweets. There are several practical reasons to leave the retweets; tracing important tweets and their dissemination is one of them. A clean version with no retweets was also released, intended for NLP researchers. We also release extracted frequent terms, bigrams, and trigrams for this community. Figure 1 outlines the steps taken to build our dataset.

As shown in Figure 1, we used SMMT to listen to the Twitter Stream API for tweets with the described keywords. We then gathered all the tweets that had the desired keywords before aggregating them locally. Our contributors used a similar procedure to gather their tweets and provided us with tab delimited files with their data. We processed them to fit our own local format to be able to include them in our dataset after deduplication (removal of tweets we have in common) and only keep unique tweet identifiers between the datasets. Table 1 represents the monthly number of tweets included in this dataset.

We then preprocessed the large set of tweets to extract the shareable meta-data of the full dataset (tweet_id, collected date, collected time), preparing the full_dataset.tsv.gz file. At the same time, we also removed tweets that were retweeted (this is, existing tweets that were re-shared by others) to create the full_dataset-clean.tsv.gz file. Our preprocessing involved cleaning up special characters, such as carriage returns, removing urls and large blank spaces. Our preprocessing was rather relaxed as we left all available languages intact. To generate the frequent terms and ngrams (sets of n-terms that appear constantly together), we removed all stop words in English and Spanish, using Spacy [24]. These lists are originally quite large, so we only shared the top 1000 terms, bigrams, and trigrams. We continued to update our original dataset every two days [14] with major releases every week [18,21] and plan to continue doing this for at least the next 12 months, a period that will likely cover the main period of the pandemic.

### Data Availability and Usage Description

The dataset is available through Zenodo [18]. There are 7 files in this repository. Table 2 details the files, formats, and their utility. The example column consists of a sample line from the files. The tweet IDs in the dataset can be hydrated using SMMT. The hydrated tweets would produce a JSON object for each tweet ID. It is important to note that when users remove their accounts or individual tweets, these are removed and are no longer available for download. In such cases, we can share the data on request while adhering to the Twitter data sharing policy. The frequent terms, bigrams, and trigrams are retrieved from the cleaned version of the dataset. The full_dataset.tsv consists of all the procured tweet IDs. The full_dataset-clean.tsv contains only original tweets with no retweets. While some applications and questions are better served with the full dataset, NLP researchers might prefer a clean dataset to have less inflated counts of the n-grams identified.

In order to use our resource, we provided all the software tools we utilized to preprocess, clean and parse the Twitter data on our Github repository [15] under the processing code directory. Note that the tweets need to be hydrated first using tools such as the Social Media Mining Toolkit or twarc [19,20]. Once the tweets are hydrated and a JSON object has been returned, we use the files parse_json_extreme.py and parse_json_extreme_clean.py to extract the tweet identifier, date of creation, text, language and a few other extra fields. This process can be configured by adding which fields from the tweet json object the user wants to extract in the fields.py file. These utilities produce a full and a clean version of the dataset, respectively, on a tab delimited file. This process is optimized to read large files without loading them fully in memory. If the user has a system with very large amounts of RAM memory, we also provide parse_json_lite.py to perform the same task. Once the JSON object has been parsed, most users will be able to directly operate on the tweets this way. We additionally provide the get_1grams.py and get_ngrams.py utilities to generate the most frequent terms and bigrams and trigrams, respectively. As the hydrated tweet JSON objects are typically quite large, we recommend separating them into daily batches to be able to more efficiently process them. All our previously mentioned tools take a single file as an input parameter for processing and output a new file. In order to combine the results of the ngram generation from multiple files, we proved the following tools that take a folder path as input and iterate through all files present: combine1grams.py, combineNgrams.py. In order to share the tweet identifiers with other groups, we provide the getDataset.py, getDataset_clean.py files which generate the equivalent files of full_dataset.tsv and full_dataset-clean.tsv that are presented in this resource in a compressed (zip) manner. Dataset statistics can be calculated with getStats.py by passing the full or clean dataset filename to them. Additionally, we released a Jupyter Notebook tutorial for novice users to include all steps to use the dataset (COVID_19_dataset_Tutorial.ipynb).

## 3. Results and Discussion

The reception of this dataset has been extraordinary, with over 119,271 downloads and over 65 citations to the preprint [16], and over 40 citations to both the Zenodo general repository and individual versions of the resource. Parts of the dataset have been used for exploratory research challenges during international NLP conferences, such as the Social Media Mining for Health (SMM4H) shared task at the North American Chapter of the Association for Computational Linguistics (NAACL) 2021 conference. Additionally, several interesting usages, particularly as an additional data source for epidemiological research [25,26], will be outlined in this section. 

### 3.1. A Google–Wikipedia–Twitter Model as a Leading Indicator of the Numbers of Coronavirus Deaths 

In [27], the authors utilize the number of Google searches, tweets from Twitter, and Wikipedia page views to determine a model of the number of people in the USA who will become infected and die from the coronavirus. To obtain the tweets from Twitter, the authors utilized version 4 of the dataset. The Google search was a leading indicator, especially for the death by state and number of cases model. The intention of this research was to develop a model which can be utilized for any epidemiological research in the future.

### 3.2. Analysis of Twitter Data Using Evolutionary Clustering during the COVID-19 Pandemic 

The authors of [28] utilize the first version of the COVID-19 dataset consisting of ~40 million tweets. The authors analyzed the tweets between 22 March and 30 March to observe the trend of public attention given to the topics related to the COVID-19 epidemic using evolutionary clustering analysis. The results indicated that unigram terms were trending more frequently on Twitter than bigram and trigram terms. Important findings from this paper include the emotional perception and sentiments of people during the COVID-19 pandemic and lockdown. Common sentiments included the fear of infection and fear of death for those who are infected. In the beginning of the pandemic, people supported lockdown measures.

### 3.3. Understanding the Public Discussion about the Centers for Disease Control and Prevention during the COVID-19 Pandemic Using Twitter Data: Text Mining Analysis Study 

This research [29] explored public sentiments about the Center for Disease Control (CDC) during the COVID-19 pandemic and was published in the *Journal of Medical Internet Research*. The authors utilized version 32 of the clean dataset containing over ~182 million tweets. This research identified 16 topics that the public linked to the CDC when they tweeted about COVID-19, which included the credibility of the CDC, policy and government response guidelines, etc. This research expresses that by efficiently identifying the topics within the public discussion on Twitter, public health agencies could improve direct communication with the public.

### 3.4. Public Risk Perception and Emotion on Twitter during the COVID-19 Pandemic 

Social science research has been published in the *Applied Network Science Journal* in which the authors presented evidence of psychological numbing [30] as the pandemic progressed. In this work [31] utilized version 14 of the dataset which contains over 400 million tweets. The authors filtered and analyzed the data for 12 countries and two languages (English and Spanish). This research demonstrates that Twitter users increasingly fixate on mortality, but in a decreasingly emotional and increasingly analytic tone. This research could potentially help policy makers and crisis management officials to understand the public attitude towards a crisis and shape their policies or announcements to balance the public perception.

### 3.5. COVID-19 Twitter Monitor: Aggregating and Visualizing COVID-19-Related Trends in Social Media 

This work [32] presents a web application, built using version 14 of the dataset which contains over 400 million tweets for COVID-19 trend visualization. The authors utilized several NLP methods such as topic modeling and sentiment analysis to aggregate the data and present the results which are easy to comprehend. The interactive plots help understand several topics associated with each topic and 30 frequent relevant terms linking to each topic. The plots also display the sentiment by hashtag and the drug brand name distribution in the dataset. Their paper was published in *ACL Anthology* and the authors demonstrated that the underlying connections in the data can be displayed by combining visualizations obtained from different methods.

### 3.6. Using Tweets to Understand How COVID-19-Related Health Beliefs Are Affected in the Age of Social Media: Twitter Data Analysis Study 

As COVID-19 cases soared, so did the spread of misinformation, leading several researchers to publish papers on infodemic research. Published in the *Journal of Medical Internet Research*, the authors of this infodemic research [33] analyzed health-related beliefs on Twitter corresponding to the disease, interventions, and influence of scientific and non-scientific events. Five thousand tweets were manually annotated to evaluate the machine learning models with the goal of employing a health belief model. Version 15 of the dataset was utilized containing over 424 million tweets. However, the authors utilized only English language tweets for their research. The authors conclude that the number of users tweeting about COVID-19 health beliefs was amplifying in an epidemic manner and could partially intensify the infodemic. The important findings of this research are: “there is no disparity between scientific and non-scientific events and the lack of substantial evidence for the speeches/tweets could be misleading”. This study helps understand the spread of misinformation during an epidemic. 

### 3.7. Changes of Diurnal Rhythms of Social Media Activities during the COVID-19 Pandemic 

This public health research [34] analyzed how social confinement affects people’s circadian rhythms at the population level. This research utilized version 20 of the dataset which has data from 1 January 2020 to 25 July 2020 with over 563 million tweets. This dataset was well utilized in this research since the authors compared social activities on Twitter for three different stages during the COVID-19 pandemic, i.e., before stay at home orders, during stay at home orders, and post stay at home orders. This research establishes the impact on people’s daily circadian rhythms captured on Twitter due to stay at home orders.

### 3.8. Characterizing Public Emotions and Sentiments in COVID-19 Environment: A Case Study of India 

Published in the *Journal of Human Behavior in the Social Environment*, this social science research [35] utilized our version 8 of the dataset with ~255 million tweets and used tweets between 22 March and 21 April 2020. The authors explored the sentiments and emotions of people in India regarding the COVID-19 pandemic. This research is an extended version of a previous similar study [36] which concluded that the sentiments in India are more positive compared to the rest of the world. The authors utilized a larger dataset and observed that at the individual tweet level, positive trends are similar to negative trends. Additionally, the authors utilized LDA models to determine the contexts of expressions while tweeting either positive or negative sentiments during the COVID-19 pandemic in India.

### 3.9. Characterizing Drug Mentions in COVID-19 Twitter Chatter 

A pharmacovigilance study which utilized version 15 of the dataset containing over 93 million clean tweets with no retweets [9] and was published in the Proceedings of the 1st Workshop on NLP for COVID-19 (Part 2) at EMNLP 2020. This study used several NLP and machine learning methods alongside traditional automated methods to recover additional data from Twitter which were otherwise lost when using only keyword-based techniques to retrieve data. The authors could obtain an additional 15% data by considering misspellings since people on Twitter tend to misspell. 

### 3.10. Large-Scale, Language-Agnostic Discourse Classification of Tweets during COVID-19 

To understand the characteristics of public attention during a crisis, the author proposed a language agnostic tweet representation to perform large-scale Twitter discourse classification with machine learning in a social science research study published in the *Machine Learning and Knowledge Extraction* journal [37]. Version 5 of the dataset was utilized in this research, which contains over 150 million tweets. This research illustrates that the large-scale surveillance of public discourse is feasible with computationally lightweight classifiers by the out-of-the-box utilization of language agnostic representations.

## 4. Conclusions

The resource presented in this work has shown great usability potential during the COVID-19 pandemic. With a wide range of applications, it is vital for such resources to be published for maximum exposure and to benefit additional researchers in communities that might not have already adopted the use of Social Media data. We showed the impact this work has had on other researchers’ work and the potential it has for epidemiological researchers [25,26] as well as other communities that have directly or indirectly performed epidemiologically related research during the pandemic [9,25,30,32,33,35,37,38,39,40,41,42]. Applications related to extracting patient narratives to understand the disease progression of COVID-19 (and long COVID) from the patient perspective [43,44] are one vital product of resources such as this one. Mixed methods approaches to identify the acceptance and usage of non-pharmaceutical interventions during the pandemic is one clear type of work that a resource such as this would enable, as users clearly voice the likes and dislikes in social media outlets. Additionally, being able to characterize the public opinion in a longitudinal way allows researchers to observe which interventions have worked, which ones have not, and what differences exist between the communities (the dataset is global), to better design and improve them.

## Figures and Tables

**Table 1:** Number of COVID-19 chatter tweets in this dataset.

	Full Dataset		Clean Dataset	
Month/Year	2020	2021	2020	2021
January	6,737,966	52,655,429	1,329,483	14,379,452
February	27,666,656	32,718,733	5,886,751	9,479,306
March	111,006,589	38,992,458	21,612,183	10,685,354
April	128,048,263	46,816,335	31,661,550	10,864,230
May	120,186,704	39,077,398	31,361,965	9,298,310
June	92,566,134	22,961,987	23,410,940	6,326,726
July	97,185,376		23,595,378	
August	73,931,454		18,614,572	
September	61,120,895		15,513,125	
October	71,836,689		18,522,752	
November	47,631,306		16,155,884	
December	51,738,825		16,366,051	

**Table 2:** Details of the released COVID-19 dataset. Note that the word TAB is not found, but instead the special ‘\t’ character is used for this. We show it on the descriptions for illustrative purposes.

File Name	Description
full_dataset.tsv.gz	A zipped, tab separated file which contains all the tweet IDs in the format—Tweet ID TAB Date TAB Time TAB language TAB country_code
full_dataset-clean.tsv.gz	A zipped, tab separated file which does not contain any retweet IDs in the format—Tweet ID TAB Date Tab Time
statistics-full_dataset-clean.tsv	A tab separated file which contains counts of total tweets each day for the clean dataset in the format—Date TAB Total No of Tweet IDs
statistics-full_dataset.tsv	A tab separated file which contains counts of total tweets each day for full dataset in the format—Date TAB Total No of Tweet IDs
frequent_terms.csv	A comma separated file which contains the counts of the top 1000 frequent terms in the following format—term, Total count
frequent_bigrams.csv	A comma separated file which contains counts of top 1000 bigrams in the format—gram, Total count
frequent_trigrams.csv	A comma separated file which contains counts of top 1000 trigrams in the format—gram, Total count
emoji.zip	A zipped collection of dated files which contain the top emojis, both in text and unicode character versions, and their frequencies per day for all clean tweets
hashtag.zip	A zipped collection of dated files which contain the top hashtags and their frequencies per day for all clean tweets
mentions.zip	A zipped collection of dated files which contain the top mentions (@) and their frequencies per day for all clean tweets

## Data Availability

Dataset is available at Zenodo: https://doi.org/10.5281/zenodo.3723939 (accessed on 21 July 2021), Code to reproduce the dataset and brief data updates: https://github.com/thepanacealab/COVID19_twitter (accessed on 21 July 2021).
